# Hours Since Last Drink Is Associated With Multiple Urine Ethyl Glucuronide Cutoffs Among Individuals With Alcohol Use Disorder

**DOI:** 10.1002/dta.70134

**Published:** 2026-08-14

**Authors:** Julianne D. Jett, Mohammad Keshtkar, Emma Seidler, Katharine Palmer, Diana Tyutyunnyk, Rachael Beck, Sara C. Parent, Douglas L. Weeks, Naomi Chaytor, Sterling M. McPherson, Michael G. McDonell

**Affiliations:** ^1^ Department of Community and Behavioral Health, Elson S. Floyd College of Medicine Washington State University Spokane Washington USA

**Keywords:** alcohol, alcohol timeline followback (ATLFB), biomarker, hours, urine ethyl glucuronide (uEtG)

## Abstract

Ethyl glucuronide in urine (uEtG) is a biomarker that objectively detects alcohol consumption. However, what uEtG values represent regarding the last drinking event remains unclear. This study investigated whether the recency or amount consumed at the last event are associated with multiple uEtG cutoffs indicative of light to heavy drinking. Adults (*n* = 349) recruited for an alcohol use disorder clinical trial submitted urine samples and self‐reported alcohol measures at baseline and twice weekly during a 4‐week observation period prior to treatment. The ThermoFisher DRI immunoassay was used to quantify uEtG levels (0–2000 ng/mL). Self‐report measures included the number of hours since last drink (Hours), number of standard drinks at that event (Amount), and ratios of these measures (Hours:Amount and Amount:Hours). Three uEtG cutoffs were assessed (100, 500, and 2000 ng/mL) and generalized linear mixed modeling applied to compare self‐report measures below versus above each uEtG cutoff. A total of 1676 uEtG samples with self‐reported data were included. Comparisons across cutoffs were significant for Hours (*p* < 0.001), while Amount was only significant at the 2000 ng/mL cutoff (*p* = 0.001). Hours also had the strongest association with uEtG results across cutoffs (pseudo‐*R* ^*2*^ range = 0.474–0.530). Higher uEtG results indicated more recent drinking, rather than higher levels of alcohol consumption, and the period of detection of uEtG was shorter than previously reported. Even at the low threshold of 100 ng/mL, uEtG was best conceptualized as a measure of alcohol consumption in the prior 24 h, with higher uEtG levels being associated with fewer hours since last drink.

## Graphical abstract

Higher uEtG results indicated more recent drinking, rather than higher levels of alcohol consumption, and the period of detection of uEtG was shorter than previously reported. Even at low cutoffs, uEtG was best conceptualized as a measure of alcohol consumption in the prior 24 h.

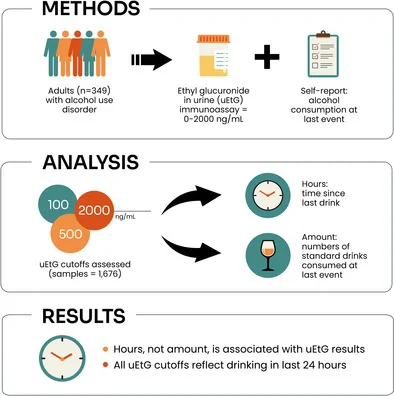

## 1 Introduction

Self‐reported alcohol use measures, like the alcohol timeline followback (ATLFB), are considered the gold standard for assessing consumption. However, stigma and poor recall can lead to underreporting, thus a combination of self‐report and objective measures of alcohol use (i.e., biomarkers) are needed to rigorously assess drinking patterns [1, 2]. The rapid elimination of ethanol from the body causes biomarkers that assess ethanol directly to have a short window of detection (e.g., breath and blood alcohol < 12 h) [3]. In contrast, ethyl glucuronide is a metabolite of ethanol that can be detected in urine, blood, saliva, hair, and nail samples after ethanol has been eliminated from the body, thereby extending the window of detection and improving identification of recent drinking [4]. Due to the ease of sample collection, ethyl glucuronide in urine (uEtG) is commonly used in research, forensic, and clinical settings to verify abstinence and assess alcohol use disorder (AUD) treatment efficacy. uEtG peaks in concentration within 4–5 h of consumption and has a half‐life of 2–3 h [5, 6, 7, 8, 9]. In clinical practice and criminal legal settings, uEtG assessment is typically limited to a single cutoff that identifies light to moderate drinking (i.e., 300–500 ng/mL) [7, 10, 11, 12]. Characterizing a wider range of uEtG cutoffs, in terms of the recency and amount of drinking needed, would provide valuable information to clinicians, courts, and others who use uEtG to assess alcohol consumption and direct related healthcare.

Previously, a low uEtG cutoff of 100 ng/mL was found to detect 77% of light drinking events 48 h prior to urine collection and 79% of heavy drinking events (≥ 4 standard drinks in one drinking event) up to 120 h prior to urine collection [13]. In another study, 72% of those who self‐reported drinking within 24 h of sample collection were positive on a uEtG point‐of‐care test (≥ 300 ng/mL), with proportions of positive tests decreasing to 28% when drinking occurred 48–72 h prior [11]. Time since last drink was found to be the greatest predictor of a positive uEtG test. However, alcohol consumption was assessed daily rather than hourly, thus limiting clinical interpretation of how the drinking event most proximal to testing impacts uEtG results. Additionally, the point‐of‐care test used in the study only assessed a single cutoff that is associated with no consumption to light consumption. Whether the relationship between recency or amount of drinking and uEtG results changes following a heavy drinking event remains unclear. A continuous measure that can report on a wider range of uEtG limits is needed to more precisely characterize the relationship between recency (hours) and/or amount of drinking and uEtG results, particularly among people who engage in high levels of alcohol consumption (uEtG 500–2000 ng/mL).

The goal of the present study was to further clinical interpretation of what uEtG results represent by (1) capturing self‐reported measures specific to the last drinking event and (2) using a continuous uEtG measure to assess multiple cutoffs representative of light to heavy drinking (0–2000 ng/mL). Specifically, we investigated the association between uEtG cutoffs and the number of hours since the last drink (Hours) and the number of standard drinks consumed at the last event (Amount). It is possible that the relationship between Hours and Amount (e.g., number of standard drinks per hour) has a stronger association with uEtG values than either self‐report measure alone; thus we also evaluated ratios of these self‐reported measures of alcohol consumption.

## 2 Methods

### 2.1 Participants

Participants were adults recruited for an outpatient randomized controlled trial that evaluated the efficacy of contingency management interventions for alcohol consumption. Participants met inclusion criteria at the baseline visit if they (1) were 18–65 years of age, (2) spoke English, and (3) self‐reported five heavy drinking episodes in the last 30 days (≥ 4 standard drinks for females assigned at birth, ≥ 5 standard drinks for males assigned at birth). Participants were excluded if they had (1) an inability to provide consent as assessed by the Macarthur Competence Assessment Tool for clinical research [14], (2) a severe substance use disorder as assessed by the Diagnostic and Statistical Manual Structured Clinical Interview for the DSM‐5 (SCID‐5, excluding cannabis) with self‐reported drug use in the past 30 days or a positive urine drug screen at baseline (3) a history of medical conditions associated with risk of life‐threatening alcohol withdrawal (e.g., seizure disorders), or (4) plans to become pregnant or currently pregnant. Participants provided written informed consent to participate in the study. Study procedures were approved by Washington State University's Institutional Review Board (16326) and registered at Clinicaltrials.gov (NCT03481049).

### 2.2 Study Procedures

Alcohol use measures (i.e., uEtG and self‐report) were collected in‐person at baseline and twice weekly during the 4‐week observation period prior to treatment. Participants received compensation for providing urine samples regardless of their uEtG result. Up to nine visits were completed per participant (one baseline and eight observation visits).

#### 2.2.1 Self‐Reported Alcohol Consumption

The ATLFB measure was adapted to include the number of hours since the last drinking event (Hours) and the number of standard drinks consumed at that event (Amount) [11]. Self‐reported measures reflected drinking for up to 120 h (i.e., 5 days) prior to the study visit per previous studies [13]. In addition to Hours and Amount, we assessed ratios of these variables (i.e., Hours:Amount and Amount:Hours) to determine if accounting for both parameters simultaneously improved prediction of self‐reported alcohol outcomes. A larger Hours:Amount ratio would indicate that more time had passed with fewer drinks consumed at the last drinking event, while a larger Amount:Hours ratio would indicate a higher number of drinks were consumed within a shorter duration of time since the last drinking event.

#### 2.2.2 uEtG Immunoassay

At each visit, urine samples were self‐collected by participants and the authenticity of the specimen verified by staff (i.e., specific gravity and creatinine; iScreen Specimen Validity Test). Urine samples were then analyzed onsite for uEtG using the Indiko DRI benchtop immunoassay (ThermoFischer Scientific, Freemont, CA). The analyzer was calibrated weekly for uEtG (0–2000 ng/mL) and quality controls conducted daily. Any value identified as below or above the limit of detection was recorded as 0 or 2000 ng/mL, respectively. For the purposes of characterizing alcohol consumption, three uEtG cutoffs were assessed: 100, 500, and 2000 ng/mL. Cutoffs were based on prior validation studies or represented the highest level of detection for the immunoassay [10, 12, 13]. For each cutoff, uEtG results were assigned a binary value to indicate whether the sample was less than each cutoff versus greater than or equal to each cutoff (0 or 1, respectively).

#### 2.2.3 Data Analysis

Generalized linear mixed modeling (LMM) was used to evaluate differences among samples below each uEtG cutoff to samples at or above each uEtG cutoff via group main effects. The LMM framework accommodated for autocorrelation among samples due to nesting of repeated individual observations within participants with more than one uEtG sample, as well as for different numbers of observations per participant. All models used restricted maximum likelihood estimation with identity link functions, random intercepts, a fixed effect for group (i.e., ≥ versus <), a fixed effect for sex assigned at birth, and a random categorical repeated measures time index (i.e., up to nine samples). Degrees of freedom for denominators in tests of main effects were approximated with the Satterthwaite approach. Initial models included age as a time‐invariant covariate; however, inclusion did not allow solution of the Hessian matrix resulting in non‐convergence. Age was eliminated from further modeling to allow models to converge. Goodness of model fit was assessed for various working correlation matrix structures; in all models, goodness of model fit was optimized with variance component correlation matrices. Model fit to the data was assessed with pseudo‐*R* ^*2*^ coefficients of determination. Separate LMMs were developed for each outcome at each cutoff. Estimated marginal means are reported for groups on either side of the established cutoff for each outcome. All analyses used two‐sided type I error rates of *p* < 0.05 and were conducted with IBM SPSS Statistics, v. 29.0 (IBM, Armonk, NY).

## 3 Results

The participant population (*n* = 349) was evenly distributed by sex assigned at birth (49.3% female), predominantly non‐Hispanic White (76.3%), and had a mean age of 40.6 years old (SD 11.5) with a greater than high school education (13.5 years, SD 3.8). Results included urine samples with self‐reported drinking within the previous 120 h. The analysis included 1676 urine samples with paired ATLFB measures, averaging to 4.8 samples per participant. Additionally, 58% of urine samples had values at or above the upper limit of detection for the uEtG immunoassay and were coded as ≥ 2000 ng/mL.

LMM detected a significant main effects for groups formed by each uEtG cutoff (i.e., 100, 500, and 2000 ng/mL) for hours since last drinking episode (Hours), the ratio of hours since last drink to number of drinks at that event (Hours:Amount), and the ratio of number of drinks to hours (Amount:Hours). For each uEtG cutoff, the means for these self‐reported outcomes were significantly larger for participants at or above each cutoff compared with those below each cutoff (all *p* < 0.01; see Table 1). Significant main effects for the number of drinks at the last event (Amount) were not detected at the 100 and 500 ng/mL cutoffs (*p* = 0.168 and 0.361, respectively); however, the main effect for the 2000 ng/mL cutoff was significant (*p* = 0.001). Furthermore, no main effects of sex assigned at birth or associated interactions with cutoffs were detected for Hours or either ratio (all *p* > 0.166). However, for Amount, individuals assigned male at birth reported significantly more drinks than females at the last drinking event (*p* < 0.001, male mean ± standard error = 5.63 + 0.23; female mean ± standard error = 4.47 ± 0.23). A coefficient of determination analysis also indicated that the strength of prediction was greatest for Hours across all uEtG cutoffs (pseudo‐*R* ^*2*^ range = 0.474–0.530) in contrast to the other self‐reported alcohol use outcomes, including Amount or either ratio (pseudo‐*R* ^*2*^ square range = 0.267–0.407, see Table 2).

**TABLE 1:** The means (SE) represent comparisons between uEtG results below each cutoff to those equal or above each cutoff. In contrast to time since last drink (Hours), the amount consumed at the last event (Amount) was only significant for the 2000 ng/mL cutoff. For each cutoff, negative and positive test results were associated with an Amount indicative of a heavy drinking event (≥ 4 standard drinks in one setting), further emphasizing the importance of Hours in differentiating test results.

Alcohol measure	100 ng/mL below/above	500 ng/mL below/above	2000 ng/mL below/above
Hours	54.47/19.77 (1.23/0.83) ***p* < 0.001**	47.77/15.56 (0.97/0.83) ***p* < 0.001**	41.50/13.49 (0.93/0.95) ***p* < 0.001**
Amount (standard drinks)	5.12/5.37 (0.29/0.21) *p* = 0.361	5.11/5.44 (0.25/0.22) *p* = 0.168	4.95/5.71 (0.23/0.24) ***p* = 0.001**
Hours:Amount ratio	22.15/6.79 (0.85/0.54) ***p* < 0.001**	19.09/4.99 (0.68/0.57) ***p* < 0.001**	16.49/3.93 (0.62/0.64) ***p* < 0.001**
Amount:Hours ratio	0.35/0.73 (0.14/0.10) ***p* = 0.009**	0.32/0.84 (0.12/0.10) ***p* < 0.001**	0.31/1.00 (0.11/0.11) ***p* < 0.001**

**TABLE 2:** Pseudo‐*R* square coefficients illustrate the strength of association between each uEtG cutoff and self‐reported measures from the last drinking event. Across cutoffs, time since last drink (Hours) had the strongest association with uEtG. Though ratios were significantly correlated with uEtG cutoffs in Table 1, the strength of those associations was found to be weaker than Hours alone.

Alcohol measure	100 ng/mL below/above	500 ng/mL below/above	2000 ng/mL below/above
Hours	0.500	0.530	0.474
Amount (standard drinks)	0.403	0.402	0.407
Hours:Amount ratio	0.317	0.331	0.313
Amount:Hours ratio	0.267	0.269	0.274

## 4 Discussion

Among individuals with AUD who self‐reported drinking in the last 120 h, we found that uEtG values were more strongly associated with time since the last drinking event (Hours) than the number of standard drinks at that event (Amount). Samples that were above the 100 ng/mL cutoff (i.e., positive) reported drinking on average 19.8 h prior to urine collection, while those at or above the 2000 ng/mL cutoff reported drinking on average 13.5 h prior to urine collection (Table 1). In contrast, the number of standard drinks required to attain a positive uEtG result at each cutoff varied little, with an average of 5.1 (100 ng/mL) to 5.7 (2000 ng/mL) standard drinks. Only the 2000 ng/mL cutoff was significantly associated with Amount. Additionally, the average number of drinks reported at each cutoff indicated a heavy drinking event occurred (≥ 4 standard drinks) for both negative and positive results, further emphasizing the importance of time when differentiating outcomes (e.g., 100 ng/mL cutoff: negative result = 5.12 standard drinks, 54 h prior; positive result = 5.37 standard drinks 19 h prior). Though combining the Hours and Amount variables into ratios was significantly associated with uEtG cutoffs, neither ratio improved the strength of the associations relative to Hours alone (Table 2). Collectively, these data have the potential to inform the interpretation of uEtG results in clinical and forensic settings regarding (1) the recency of drinking events and (2) the frequency of testing needed to reliably detect heavy drinking events.

Previously, we found that a uEtG cutoff of 100 ng/mL accurately identified a light drinking event (1–2 standard drinks) for up to 48 h and a heavy drinking event for up to 120 h [10, 13]. In contrast, the current study found that samples positive at the 100 ng/mL cutoff were associated on average with a heavy drinking event in the previous 24 h. A strength of the present study that may contribute to differences in the uEtG detection window, relative to Lowe et al., is having a larger number of urine samples collected (1676) and participants recruited (*n* = 349). Collecting urine samples during an outpatient observation period (i.e., consuming per usual) and having a relatively even distribution of sex assigned at birth, likewise increases the generalizability of our findings. Another strength of the current study is the utilization of self‐reported measures that focus on the last drinking event (i.e., hours, rather than days), which is more temporally and clinically relevant for the interpretation of uEtG values given the metabolite's half‐life [7]. Furthermore, using an analytical method with a large uEtG detection window (0–2000 ng/mL) enabled us to assess the relationship between self‐reported measures and multiple uEtG cutoffs rather than a single cutoff, as found with point‐of‐care tests.

It is important to note that participants in this study were treatment‐seeking and predominantly met criteria for severe AUD (68%, DSM‐5 symptom score of 6–11). Thus, the relationship between uEtG and Hours or Amount may differ for individuals who have less severe forms of AUD [7, 15]. Additionally, there was no main effect of sex assigned at birth on the relationship between uEtG cutoffs and self‐reported measures (*p* ≥ 0.166); however, our analyses did not account for other individual factors that could theoretically impact uEtG levels, such as medications, diet, and body mass index. Interpretation of results should also consider limitations imposed by the measures used. Notably, 58% of urine samples analyzed were above the immunoassay's upper limit of detection (≥ 2000 ng/mL), which restricted our ability to assess the relationship between uEtG and self‐report measures following heavier drinking events. Our reliance on self‐reported measures to characterize uEtG results is also a limitation of the current study. Self‐reported measures are susceptible to poor recall and inaccuracies with reporting the number of standard drinks [1, 2]. Focusing measures on the last drinking event, rather than recalling consumption daily or for multiple days, is likely to reduce error caused by poor recall though this adaptation to the ATLFB has not been validated. Alternatively, employing additional alcohol biomarkers (e.g., transdermal monitoring) or self‐reporting drinking events in real‐time with a drinking diary would help to objectively validate the last drinking event and further inform uEtG values.

Our findings from the current study indicate that the number of hours since the last drinking event, not the amount of alcohol consumed at that event, differentiates uEtG cutoffs. Even at low cutoffs (e.g., 100 ng/mL), positive uEtG values are associated with heavy drinking events in the previous 24 h, suggesting that daily uEtG testing may be needed to accurately identify drinking events. Future studies are needed to investigate the relationship between these parameters in samples from individuals with no AUD and mild AUD, as well as non‐treatment seeking individuals. Likewise, the current study presents group means which can vary from individual to individual. Investigating the association between uEtG and self‐reported measures longitudinally within‐subjects would inform how different drinking patterns (e.g., binge drinking versus daily heavy drinking) and health variables impact uEtG results. Lastly, studies utilizing analytical methods with a higher uEtG level of detection (i.e., liquid chromatography‐mass spectrometry) are needed to determine the association between Hours and Amount in samples with uEtG values that exceed our immunoassays' upper limit of detection (i.e., > 2000 ng/mL).

## Funding

This study was supported by the National Institute on Alcohol Abuse and Alcoholism (R01AA020248, PI: Michael G. McDonell; K01AA032054, PI: Julianne D. Jett).

## Conflicts of Interest

The authors declare no conflicts of interest.

## Data Availability

The data that supports the findings of this study are available from the corresponding author upon request.
